# Assessing Potentially Inappropriate Medications in Nursing Home Residents by NORGEP-NH Criteria

**DOI:** 10.3390/pharmacy7010026

**Published:** 2019-03-05

**Authors:** Kjell H. Halvorsen, Sinan Kucukcelik, Beate H. Garcia, Kristian Svendsen

**Affiliations:** 1 Department of Pharmacy, Faculty of Health Sciences, UiT The Arctic University of Norway, Tromsø N-9037, Norway; sinan.kc7@gmail.com (S.K.); beate.garcia@uit.no (B.H.G.); kristian.svendsen@uit.no (K.S.); 2 Hospital Pharmacy of North Norway Trust (Sykehusapotek Nord HF), Tromsø N-9291, Norway

**Keywords:** nursing home residents, potentially inappropriate medications, quality of health, pharmacoepidemiology, drug therapy, explicit criteria lists

## Abstract

**Background:** Nursing home residents often have several conditions that necessitate the use of multiple medicines. This study investigates the prevalence of potentially inappropriate medications (PIMs) and its associations with sex, age, number of medicines, and study location (rural/urban). **Methods:** A cross-sectional study of long-term care residents from six nursing homes. Data was collected from medical records. We identified PIMs by applying the NORGEP-NH criteria. We conducted a Poisson regression analysis to investigate the association between the number of PIMs and sex, age, number of medicines, and study location. **Results:** We included 103 (18.4%) of 559 residents (68.0% women; mean age 83.2 years, mean number of daily used medicines 7.2 (SD = 3.6)). We identified PIMs in 56% of the residents (mean number = 1.10, SD = 1.26). In adjusted analyses, residents ≥80 years had 0.43 fewer PIMs compared to residents <80 years (*p* < 0.05). Residents using 4–6, 7–9, and 10+ medicines had on average 0.73, 1.06, and 2.11 more PIMs compared to residents using 0–3 medicines (*p* < 0.001), respectively. **Conclusion:** PIM use is prevalent among nursing home residents and is significantly associated with age and number of medicines. Our findings suggest a modest decrease in residents using PIMs compared to previous studies. Nevertheless, prescribing quality in nursing home residents in both urban and rural areas is still of great concern.

## 1. Introduction

Nursing homes in Norway provide long-term care to more than 35,000 residents, and more than 80% of the residents suffer from cognitive impairment and have many comorbidities [1]. Multiple comorbidities often necessitate the use of multiple medicines [2], but providing optimal medicine therapy is challenging due to age-related physiological changes [3]. Complex medication regimens and polypharmacy expose patients to drug-related problems like drug–drug interactions and adverse drug reactions [4]. In addition, the risk of medicine-induced cognitive impairment, e.g., due to additive anticholinergic properties, is essential to take into consideration [5]. Due to health deterioration among these residents during their later years, the use of medicines increases substantially, which again raises the risk of prescribing potentially inappropriate medications (PIMs) [6].

PIMs are defined as “medications that have more potential risk than potential benefit or prescribing that does not agree with accepted medical standards” [7]. Different strategies exist to identify PIMs among older patients. The application of criteria-based screening tools is a broadly applied strategy. The use of such tools helps clinicians to identify specific medicines or drug classes, combinations of different medicines, or dosages that may cause harmful effects among older patients. The first such tool for assessing PIMs in older patients was the Beers list published in the USA in 1991 and has later been updated several times [8,9]. National differences in drug markets and prescribing traditions have led to the development of several country-specific tools, e.g., the French consensus panel list of PIMs in older persons [10] and the Irish Screening Tool of Older Persons’ Potentially Inappropriate Prescriptions (STOPP) [11]. In Norway, the Norwegian General Practice Nursing Home (NORGEP-NH) criteria was published in 2015 [12,13].

Recent studies applying screening tools suggest that PIMs are frequent among nursing home residents [3,14,15,16]. A systematic review investigating the point prevalence of PIM use in 227,534 nursing home residents ≥60 years based on 26 studies found a considerably higher PIM use after 2005 compared to 1990–1999 (49.8% vs. 30.3%). The review also reported that PIM use was higher among nursing home residents in Europe compared to those in North America (49.0% vs. 26.8%) [17]. Similarly, the use of PIMs among Norwegian nursing home residents has also increased from 1993 to 2011, for single substances to avoid (36.8 vs. 39.5%), for combinations to avoid (16.3 vs. 27.0%), and for deprescribing items (46.0 vs. 55.3%) [6]. The majority of studies report that the total number of prescribed medications is the most important factor associated with use of PIMs [17]. Due to an increased focus on the quality of prescribing in nursing homes during the last decade and a lack of recently published data on PIMs, we aimed to investigate the prevalence of PIMs among nursing home residents applying the most recently published Norwegian screening tool for PIMs. We also aimed to explore whether the factors sex, age, number of medicines and study location (rural/urban) were associated with the use of PIMs.

## 2. Materials and Methods

### 2.1. Study Setting and Population

This cross-sectional study recruited nursing home residents from an urban and a rural area in North Norway. In the urban area, we recruited from four out of five nursing homes (total long-term beds = 320), i.e., those nursing homes that accepted our request to perform the study. In the rural area, we recruited from two out of six nursing homes (total long-term beds = 239). These two nursing homes were chosen based on the number of residents, located in two different municipalities, and within acceptable travel distance for the data collector.

### 2.2. Inclusion and Exclusion

We included only long-term nursing home residents, which are persons with no expectations of returning to assisted living at their homes. Written consent from the resident or the resident’s next of kin was necessary in order to collect data. Nurses at the respective nursing homes facilitated the patient recruitment and collection of consents. There were no exclusion criteria.

### 2.3. Data Collection

Three pharmacists (BPharm) collected data from electronic medical records and medicine charts at the nursing homes from November 2015 to January 2016. For each resident, the following variables were obtained: sex, age, medical diagnoses, prescribed medicines (drug name, strength, formulation, dosage, and whether used regularly or as needed). All data was recorded anonymously on an Excel^®^ spreadsheet (Microsoft Windows, Redmond, WA, USA), i.e., patient identity was replaced by consecutive running numbers. All medicines were coded according to the Anatomical Therapeutic Chemical (ATC) Classification System [18]. The ATC classification system classifies the active substances in a hierarchy with five different levels. In this study, we report data on the third level which describe chemical, pharmacological, or therapeutic subgroups level, and the 5th level which denotes the chemical substance or the generic name, e.g., oxazepam. 

### 2.4. Identification of PIMs

To identify PIMS, we applied the NORGEP-NH criteria tool comprising 34 criteria divided into three sub-domains: 11 single-drug criteria, 15 drug–drug combination criteria, and 8 criteria where regular consideration of deprescribing or dose adjustments should be considered [13]. We excluded criterion 31 and 34, both deprescribing criteria, concerning medicines lowering blood pressure and preventive medicines, as the criteria are unspecific with regard to which medicine to assess. 

### 2.5. Statistical Analysis

Results are presented as numbers (n), proportions (%), and means with standard deviation (SD). We performed the analysis using STATA statistical program version 13.1 by importing data from the Excel^®^ spreadsheet [19]. Student’s *t*-test was applied to compare means (continuous data; age, number of medicines used). Fisher´s exact test was used to compare proportions (categorical data; sec, study location). We considered p-values <0.05 statistically significant.

We conducted a multiple Poisson regression analysis to study the association between number of PIMs and sex (dichotomous; female/male), age (dichotomous; under 80 years/80+ years), location (dichotomous; urban/rural), and the number of medicines used on a regular basis (categorical; 0–3 medicines, 4–6 medicines, 7–9, 10+ medicines). Results presented as marginal effects represent the average change in the number of PIMs that is explicitly associated with one unit or category change in the value of the independent variables.

### 2.6. Ethics and Approvals

The Regional Committee for Medical and Health Research Ethics had no objections to the study. The Norwegian Center for Research Data approved the study. 

## 3. Results

During the study period, we approached 125 (22.3%) of 559 nursing home residents. A total of 103 (18.4%) residents provided informed consent (68.0% women; mean age 83.2 years). Table 1 summarizes resident characteristics. 

### 3.1. Diagnoses and Medicine Use

The five most commonly registered diagnoses were dementia (41%), hypertension (31%), atrial fibrillation (15%), diabetes (15%), and osteoporosis (14%). The mean number of medicines used on a regular basis and as needed was 7.2 (SD = 3.6) and 3.7 (SD = 1.9), respectively, differing significantly between the urban and rural nursing homes (*p* < 0.001), as shown in Table 1.

Six drug classes (ATC third level) were used by more than 40% of the residents and included analgesics and antipyretics (89.3% of the residents), medicines for constipation (72.8%), antithrombotic agents (49.5%), opioids (47.6%), hypnotics and sedatives (45.6%), and anxiolytics (40.8%), as shown in Table 2.

### 3.2. PIMs Identified

We identified 113 PIMs related to regular medication use in 103 residents, a mean number of 1.10 (SD = 1.26) PIM per resident. We identified at least one PIM in 57% (n = 5), at least two PIMs in 30% (n = 31), and at least three PIMs in 16% (n = 16) of the residents (Poisson distribution). We identified no difference between women and men or between study locations. 

For nine of the 32 applied criteria, we identified no PIMs. The identified PIMs concerned 29 single medicines, 16 medicine combinations, and 46 deprescribing or dose-adjustment medicines (Table 3). Use of chlomethiazole (5.8% of residents) and regular use of hypnotics (23.3%) were the two most common single medicine criteria. Among combinations to avoid, concomitant use of three or more psychotropic medicines was identified in 7.8% of the residents, with no significant differences between study locations (*p* = 0.265). Apart from warfarin in combination with SSRI/SNRI, most other criteria within this domain were barely detectable. Concerning the deprescribing criteria, 26.2% of the residents used antidepressants, while 15.5% used antipsychotics. Significant differences between study location related only to the use of bisphosphonates (*p* = 0.009). 

### 3.3. Factors Associated with the Use of PIMs

Table 4 presents the results of the Poisson regression analysis. The analysis revealed that residents ≥80 years had 0.43 fewer PIMs compared to residents <80 years (*p* < 0.05, pseudo R^2^ = 0.16.). The number of PIMs identified was significantly associated with the number of medicines used in the following way; in residents using 4–6, 7–9, and 10+ medicines, we identified on average 0.73, 1.06, and 2.11 more PIMs compared to residents using 0–3 medicines, respectively (*p* < 0.001). Neither sex nor study location (urban vs. rural) were associated with the number of PIMs identified.

## 4. Discussion

This study confirmed that medication use and PIM use among nursing home residents is extensive. However, we did not identify any significant associations between PIM use and sex or study location (urban/rural). As expected, we found a significant association between PIMs and the number of medications. We also identified a significantly lower number of PIMs in residents 80 years or older. 

PIMs were identified among 57% of the 103 included nursing home residents. This is lower compared to findings by Nyborg et al. reporting that 70% of Norwegian nursing home residents in Vestfold used PIMs between 2009–2011 [20], but higher than numbers from Canada (48%) [21] and in line with results from Ireland (60%) [15]. As patient characteristics are comparable to former Norwegian studies, a patient selection bias cannot entirely explain the difference in our findings. Also, the mean number of medicines used on a regular basis in the present study seems to be slightly higher compared to previous studies: 6.9 medicines in 2011 [5] compared to 7.2 in our study, which normally would lead to a higher proportion of PIMs [16]. However, this was not observed in this study. Our findings based on more recent data may reflect a real shift in prescribing behavior among nursing home physicians. This may be due to the increased focus on reducing inappropriate prescribing in Norwegian nursing homes during recent years [6,20,22], but needs to be confirmed by other and larger studies.

### 4.1. Use of Medicines Related to the Central Nervous System and Risk of Falls

Of the ten medicine groups most frequently used by the nursing home residents, five were used to treat diseases and symptoms related to the central nervous system, i.e., pain, anxiety, sleeping disorders, and episodes of depression (Table 2). The significant proportion of psychotropic medicines and the complex medication regimes in these residents call for special attention when it comes to preventing adverse drug events. In particular, the risk of falls may be increased in this population as six of the medicine groups (denoted * in Table 2) are considered to increase fall risk directly or may cause or worsen orthostatic blood pressure [23]. Milos et al. reported that fallers among nursing home residents used on average a higher number of these medicines compared to non-fallers [24]. Furthermore, other studies report that patients taking four or more regular medications have a significant increased risk of falling [25].

### 4.2. PIMs Related to the Use of Psychotropics

The four most frequently identified PIM criteria in this study were related to medications with effect on the central nervous system, i.e., “regular use of hypnotics” (criterion 11), “concomitant use of three or more psychotropics” (criterion 23), “antipsychotics (deprescribing)” (criterion 27), and “antidepressants (deprescribing)” (criterion 28), as shown in Table 3. This may reflect the nature of the NORGEP-NH criteria, focusing mostly on these medications [13]. Compared to previous Norwegian studies from nursing homes, the prevalence rates of the four abovementioned criteria have changed from 2011 to 2016, from 30.4 to 23.3%, 19.9 to 7.8%, 15.7 to 15.5%, and 35.8 to 26.2%, respectively [6]. The reduction observed for three of the criteria may indicate that physicians have become more aware of the PIM medicines included in the NORGEP list and the potential problems associated with these. In addition, other health care professionals like nurses and pharmacists may have contributed to the increased focus on optimizing medicine use among nursing home residents through the Norwegian Patient Safety Program: In Safe Hands [26]. We found an unexpected reduction of PIMs related to the criterion ”concomitant use of three or more psychotropics”. There are reports of an increasing use of opioids, pregabalin, and gabapentin in the treatment of pain among nursing home residents, as these medicines are believed to more efficiently reduce behavioral disturbances [27]. 

### 4.3. Factors Associated with the Number of PIMs Identified

We identified no significant difference between sex or study location, but the number of PIMs identified was significantly associated with the number of medicines used (Table 4). This finding is consistent with previous studies [15,28]. Interestingly, we identified significantly fewer PIMs among residents aged 80+ compared to those below 80, which is opposite to what most other nursing home studies have found [17,29]. An Irish study (n = 133) by Parson et al., evaluating PIMs using STOPP criteria in older patients with dementia in care homes, did not report any association between PIMs and age [29], neither did a Norwegian study assessing PIMs in nursing home residents (n = 881) using NORGEP-NH [17]. Our findings may be prone to selection bias because of a small study population or by our decision to not include medicines taken as needed in the analyses. On the other side, our findings may reflect a real shift in prescribing behavior among nursing home physicians as described above.

### 4.4. Strategies to Combat Use of PIMs

This study confirms the necessity to develop a strategy that can reduce PIMs among nursing home residents. Most likely, such a strategy needs to be multifaceted and include multicomponent interventions. The knowledge translation strategy for hospitalized elderly adults comprising the distribution of educational materials, presentations by geriatricians, pharmacist–physician interventions based on alerts from a computerized alert system, and comprehensive geriatric assessments have been found to be effective in reducing PIMs in other settings and might serve as a template to develop such a strategy also in the nursing home setting [30]. In addition, the significant correlation between the total number of medicines used and PIMs identified [31], and the fact that three out of four nursing home residents still are prescribed curative or preventive medicines on the day they die [32], indicate that clinicians and clinical pharmacists should to a wider extent focus on deprescribing in these residents. 

All deprescribing efforts, i.e., to taper and then discontinue medicines must be carefully planned and monitored. Tapering and discontinuation of medicines may be considered in different scenarios, for instance, when the condition is no longer present, when the medicine is considered inappropriate, if the medicine causes more harm than benefit, when there is uncertainty about the actual effect of the medicine, or in situations where the residents themselves are unwilling to continue drug treatment. In any of these situations, it is essential to monitor the process of tapering and/or discontinuation and be aware of potential withdrawal symptoms. A better understanding of possible symptoms can both prevent re-prescribing and consequent prescribing cascades. To avoid such episodes it is important to know if the medication can be abruptly discontinued or whether a reversed “start low–go slow” strategy should be followed. Fortunately, the number of ongoing research projects concerning the topic will definitely provide future guidance, as well as the establishment of national and regional deprescribing networks [33]. In addition, health care educators need to address the topic more specifically in the curricula and training of pharmacists, nurses, and physicians.

### 4.5. Strengths and Limitations

Direct comparison between studies investigating PIMs is challenging due to use of different criteria, criteria applicability, and study settings. Consequently, the prevalence of PIMs among nursing home residents varies extremely between studies. One example illustrating the variance in PIM identification is when Chen et al. assessed PIMs in 211 Malaysian nursing home residents not suffering from dementia. They identified PIMs in 23.7% of the residents applying the STOPP criteria and in 32.7% applying the Beers criteria. Adding to the huge variance in PIM identification, considerably higher prevalence rates of PIMs have been reported among residents suffering from dementia [5]. Although this may imply that residents with dementia are at higher risk of receiving PIMs, evidence is conflicting as others have reported that dementia might also be associated with a lower likelihood of using PIMs [34]. 

This study made exclusive use of medical records and medicine charts without exploring prescribers’ views or intentions to treat with PIMs. Neither did we elicit patients’ perceptions about the use of the medications appearing on the NORGEP-NH list. These are important aspects to remember for physicians, pharmacists, and nurses conducting medication reviews and assessing the use of inappropriate medications in older nursing home residents. 

The recruitment of the study population, i.e., not including residents from all the nursing homes, and the collection of point-prevalence data over two months might have introduced bias and influenced our findings. Another limitation concerns the quality of the diagnostic data in the medical journal. In the current study, only 41% of the residents had a registered dementia diagnosis, while in a longitudinal cohort study (n = 696), from nursing homes in the southeastern part of Norway, data collection revealed that 84% of the population had dementia. However, only 56% had the diagnosis registered in their medical journal [35]. The lack of concordance between residents’ actual conditions and their patient records needs to be kept in mind when trying to address the quality of medication use in this population. 

## 5. Conclusions

This study shows that the prevalence of PIMs in nursing home residents remains high, but that the regular use of hypnotics and concomitant use of three or more psychotropics and antidepressants seems to be reduced compared to previous studies. We identified no reduction in the use of antipsychotics compared to previous studies and no associations between PIMs and sex or study location (urban/rural). However, we observed a significant association between PIMs and number of medications used and a significantly lower number of PIMs in residents 80 years or older. Even if our findings suggest a modest decrease in PIM use, prescribing quality in nursing home residents remains a great concern.

## Figures and Tables

**Table 1:** Characteristics of the nursing home residents (n = 103).

Characteristics	Total		Urban		Rural		*p*-Value
**Patients, n (%)**	103	(100)	70	(68.0)	33	(32.0)	
Women	70	(68.0)	46	(66.0)	24	(72.7)	
Men	33	(32.0)	24	(34.0)	9	(27.3)	0.477
**Age in years, mean (SD)**	83.2	(9.5)	83.0	(9.9)	83.4	(8.6)	0.820
<80	32	(31.1)	20	(29)	12	(36.4)	
80+	71	(68.9)	50	(71)	21	(63.6)	0.425
**Medicine use, mean (SD)**							
Total	10.9	(4.3)	9.9	(4.1)	13.3	(3.6)	<0.001
Regular	7.2	(3.6)	6.1	(3.4)	9.6	(3.0)	<0.001
As needed	3.7	(1.9)	3.8	(1.9)	3.7	(2.0)	0.830
**Medical diagnoses, mean (SD)**	5.8	(2.2)	5.2	(2.3)	6.7	(1.7)	<0.001

**Table 2:** The ten most frequently used medicine groups (ATC third level) among the nursing home residents (n = 103).

ATC–Third Level		n	%
N02B	Other analgesics and antipyretics	92	89.3
A06A	Medicines for constipation	75	72.8
B01A	Antithrombotic agents	51	49.5
N02A	Opioids *	49	47.6
N05C	Hypnotics and sedatives *	47	45.6
N05B	Anxiolytics *	42	40.8
C03C	High-ceiling diuretics *	29	28.2
C07A	Beta blocking agents *	27	26.2
N06A	Antidepressants *	27	26.2
A02B	Medicines for peptic ulcer and gastro-esophageal reflux disease	25	24.3

ATC = Anatomical Therapeutical and Chemical Classification System, n = number. * Considered to increase fall risk directly or may cause or worsen orthostatic blood pressure (22).

**Table 3:** Use of potentially inappropriate medications according to the NORGEP-NH criteria in the nursing home residents (n = 103) in the urban and rural study locations.

	Total		Urban ^1^		Rural ^2^		*p* between Locations
NH-NORGEP Items	n = 103		n = 70		n = 33		
**A: Single substance criteria;** Regular use should be avoided	n	%	n	%	n	%	
1. Combination analgesic codeine/paracetamol ^1^	1	0.9	0	0.0	1	3.0	0.320
2. Tricyclic antidepressants (TCAs)	1	0.9	0	0.0	1	3.0	0.320
3. Non-steroidal anti-inflammatory drugs (NSAIDs)	1	0.9	0	0.0	1	3.0	0.320
4. First-generation antihistamines	2	1.9	0	0.0	2	6.1	0.101
5. Diazepam	1	0.9	1	1.4	0	0.0	1.000
6. Oxazepam (>30 mg/24 h)	1	0.9	0	0.0	1	3.0	0.320
7. Zopiclone (>5 mg/24 h) ^2^	4	3.9	1	1.4	3	9.1	0.096
8. Nitrazepam	2	1.9	0	0.0	2	6.1	0.101
9. Flunitrazepam	0	-	0	-	0	-	-
10. Chlometiazole	6	5.8	6	8.6	0	0.0	0.173
11. Regular use of hypnotics	24	23.3	16	22.9	8	24.2	0.877
Any single substances	29	28.2	18	25.7	11	33.3	0.484
**B: Combination criteria;** Combinations to avoid							
12. Warfarin + NSAIDs	0	-	0	-	0	-	-
13. Warfarin + SSRIs/SNRIs	3	2.9	0	0.0	3	9.1	0.031
14. Warfarin + ciprofloxacin/ofloxacin/erythromycin/clarithromycin	0	-	0	-	0	-	-
15. NSAIDs/coxibs + ACE inhibitors/AT2 antagonists	1	0.9	0	0.0	1	3.0	0.320
16. NSAIDs/coxibs + diuretics	0	-	0	-	0	-	-
17. NSAIDs/coxibs + glucocorticoids	0	-	0	-	0	-	-
18. NSAIDs/coxibs + SSRI/SNRIs	0	-	0	-	0	-	-
19. ACE inhibitors/AT2 antagonists + potassium or potassium-sparing diuretics	2	1.9	2	2.9	0	0.0	1.000
20. β-blocker agents + cardioselective calcium antagonists	0	-	0	-	0	-	-
21. Erythromycin or clarithromycin + statin	0	-	0	-	0	-	-
22. Bisphosphonate + proton pump inhibitors	1	0.9	0	0.0	1	3.0	0.320
23. Concomitant use of three or more psychotropics	8	7.8	4	5.7	4	12.1	0.265
24. Tramadol + SSRIs	0	-	0	-	0	-	-
25. Metoprolol + paroxetine/fluoxetine/bupropion	0	-	0	-	0	-	-
26. Metformin + ACE inhibitor/AT2 antagonists + diuretics	2	1.9	2	2.9	0	0.0	1.000
Any combinations to avoid	16	15.5	7	10.0	9	18.2	0.039
**C: Deprescribing criteria;** Need for continued use should be reassessed							
27. Antipsychotics (incl. “atypical” substances)	12	15.5	9	18.6	3	9.1	0.747
28. Antidepressants	27	26.2	16	22.9	11	33.3	0.259
29. Urologic spasmolytics	1	0.9	1	1.4	0	0.0	1.000
30. Anticholinesterase inhibitors	1	0.9	1	1.4	0	0.0	1.000
31. Medicines lowering blood pressure *	-	-	**-**	-	**-**	-	-
32. Bisphosphonates	4	3.9	0	0.0	4	12.1	0.009
33. Statins	5	4.8	1	1.4	4	12.1	0.035
34. Any preventive medicine *	-	-	-	-	-	-	-
Any deprescribing item	46	44.7	26	37.1	20	60.6	0.034
Any NORGEP-NH criteria	58	56.3	35	50.0	23	69.7	0.088

* Not applicable. ^1^ four nursing homes, ^2^ two nursing homes.

**Table 4:** Factors associated with identification of potentially inappropriate medications (PIMs) among the nursing home residents presented as marginal effects; multivariate Poisson regression.

		Poisson Regression		
		Marginal effect	95% CI	*p*-value
Sex				
	Female	Ref		
	Men	0.37	−0.12, 0.87	0.14
Study location				
	Urban	Ref		
	Rural	0.02	−0.44, 0.48	0.94
Age				
	<80 years	Ref		
	80+ years	−0.43	−1.22, −0.04	<0.05 *
Number of medicines used regularly				
	0–3	Ref		
	4–6	0.73	0.40, 1.06	<0.001 *
	7–9	1.06	0.64, 1.48	<0.001 *
	10+	2.11	1.42, 2.80	<0.001 *

* Indicates significant influence (*p* < 0.05). CI; confidence interval. Pseudo R^2^ = 0.16.
